# Experimental removals reveal dietary niche partitioning facilitates coexistence between native and introduced species

**DOI:** 10.1002/ece3.5036

**Published:** 2019-03-18

**Authors:** Jonathan J. Derbridge, John L. Koprowski

**Affiliations:** ^1^ School of Natural Resources and the Environment University of Arizona Tucson Arizona

**Keywords:** coexistence, diet, introduced species, niche, wildlife

## Abstract

1. Niche overlap between native species and ecologically similar invaders can lead to competitive exclusion of threatened native species, but if two such species also co‐occur naturally elsewhere, interactions between native and introduced populations may mirror coevolved niche partitioning that reduces competition and promotes coexistence.
2. A single, insular population of Fremont's squirrel (*Tamiasciurus fremonti*) the Mount Graham red squirrel (MGRS; *T. f. grahamensis*) in the Pinaleño Mountains, Arizona, USA, is critically endangered and resource competition with introduced Abert's squirrels (*Sciurus aberti*) may threaten its long‐term persistence. The species are naturally synoptic in other mountain sites, and both consume diets comprised primarily of conifer seeds and fungi.
3. We conducted experimental removals of introduced Abert's squirrels and used stable isotope analysis of diets before and after removals, and of diets in naturally syntopic populations to test the hypothesis that dietary niche partitioning can facilitate coexistence between native and introduced species. We also developed a novel approach to determine the influence of fluctuating food availability on carbon enrichment in consumers.
4. Mount Graham red squirrels and introduced Abert's squirrels partitioned the dietary niche similarly to naturally syntopic populations. Removals had no apparent effect. Diet of MGRS was more closely linked to availability of resources than to presence of Abert's squirrels.
5. Flexible dietary niche of introduced Abert's squirrels may have allowed them to exploit a resource opportunity in syntopy with MGRS. Variable food production of MGRS habitat may intensify competition in poor years, and territorial defense against non‐native Abert's squirrels likely imposes fitness costs on individual MGRS. Similarity in our model species’ diets may make MGRS more vulnerable to competition if climate change eliminates the advantages of larder‐hoarding. Where introduced populations of ecologically similar species are better adapted to changing conditions, they may ultimately replace southern peripheral populations of native species.

## 1 INTRODUCTION

Invasive species are permanent components of most ecosystems (Vitousek, Mooney, Lubchenco, & Melillo, 1997) and understanding the effects of competition between invaders and ecologically similar natives is important to conserve biodiversity (Davis, 2003). Unlike invasive predators, which have directly caused extinctions of native species (Sax & Gaines, 2008), competitive exclusion by invaders is challenging to quantify because it may not be directly observable and population declines may have multiple causes (Sax et al., 2007). Native wildlife population declines due to invasive species have been documented (Herbold & Moyle, 1986; Petren & Case, 1996), but competition could also lead to niche partitioning and coexistence (Davis, 2003; Shea & Chesson, 2002). The ability to predict the outcome of biological invasions would be valuable to conservation of threatened populations.

Co‐occurring ecologically similar species occupy niches that limit competition and allow syntopic populations to persist (Brown, Kotler, & Mitchell, 1994; Chesson, 2000), but species introductions impose novel ecological interactions that can lack the stabilizing niche differences necessary for coexistence (Chesson, 2000). Invaders may be superior competitors for common resources or evolve rapidly to take advantage of novel resource opportunities (Shea & Chesson, 2002) and competitively exclude native species (Mooney & Cleland, 2001; Sakai et al., 2001). For example, the absence of niche partitioning (Wauters, Gurnell, Martinoli, & Tosi, 2002) and more efficient resource use (Kenward & Holm, 1993) by invasive eastern gray squirrels (*Sciurus carolinensis*) contributed to the precipitous decline of Eurasian red squirrels (*Sciurus vulgaris*) in parts of Europe (Bertolino, Cordero di Montezemolo, Preatoni, Wauters, & Martinoli, 2014; Gurnell, Wauters, Lurz, & Tosi, 2004).

In systems with variable food production, competing native species may adapt by altering their diets and feeding strategies in order to avoid direct competition (Pulliam, 1986). In two closely related species of charr (*Salvelinus* sp.) that occur in zones of sympathy, niche partitioning varied over time, and according to species and size of individuals (Nakano, Fausch, & Kitano, 1999). Niche partitioning may also be flexible in novel interactions where an introduced species occupies habitat similar to its native range, which could promote coexistence with native competitors.

We studied a model system involving interactions between Abert's squirrels (*Sciurus aberti*) and Fremont's squirrels (*Tamiasciurus fremonti*) in Arizona, USA, where the species co‐occur both naturally and in anthropogenic syntopy. One Fremont's squirrel subspecies, the Mount Graham red squirrel (MGRS; *T. f. grahamensis*) occurs in a single, critically endangered population in the Pinaleño Mountains, an isolated mountain range surrounded by desert grassland. Abert's squirrels introduced in the 1940s (Davis & Brown, 1988) have established throughout all forested areas of the Pinaleño Mountains (Hutton et al., 2003). Elsewhere in Arizona, *T. fremonti* (hereafter, red squirrel) and Abert's squirrels are naturally syntopic in zones of overlap between preferred forest types (Ferner, 1974; Hall, 1981); red squirrels inhabit primarily mixed‐conifer and spruce‐fir forest (Smith, 1981), and Abert's squirrels are typically associated with ponderosa pine (*Pinus ponderosa*) forest (Keith, 1965). Red squirrels are small (200‐250 g) larder‐hoarders that display vigorous movements and vocalizations in defending territories (Koprowski, 2005; Smith, 1968a) whereas the larger (500–750 g) Abert's squirrels are secretive, occasional scatter‐hoarders (Allred, 2010; Keith, 1965).

A potentially important effect of interaction is through competition for food. Diets of Abert's and red squirrels consist primarily of conifer seeds and fungi (Nash & Seaman, 1977; Steele, 1998). Both species also consume pollen from staminate cones of conifers during late spring and early summer (Keith, 1965; Smith, 1968a). During periods of scarcity, Abert's squirrels also depend on phloem stripped from conifer twigs (Allred, 2010; Patton, 1974), and red squirrels rely on food cached in their larder‐hoards or middens (Steele, 1998). Such divergent strategies, in addition to differential use of available food in periods of relative abundance may facilitate coexistence in natural syntopy (Chesson, 1994). As Abert's squirrel are 2–3 times larger than red squirrels, their ability to depress availability of diet items could have negative effects on MGRS especially during years of low food production. It is unknown, to what extent dietary resource partitioning occurs in the invaded community of the Pinaleño Mountains. However, the source population for Abert's squirrel translocations was naturally syntopic with red squirrels (Davis & Brown, 1988), and niche separation may be maintained by coevolved differences in habitat selection (Rosenzweig, 1981).

To examine the potential for coexistence between ecologically similar native and introduced species, we combined an experimental removal of introduced Abert's squirrels with a stable‐isotopic study of dietary niche partitioning among naturally syntopic red squirrels and Abert's squirrels. Stable isotope analysis is a well‐established method for understanding dietary resource partitioning among carnivores (Merkle, Polfus, Derbridge, & Heinemeyer, 2017) and herbivores (Bovendorp, Libardi, Saramento, Camargo, & Percequillo, 2017). We tested the hypothesis that dietary niche partitioning facilitated coexistence. We predicted: (a) low overlap in proportions of common items in MGRS and Abert's squirrel diets; (b) Abert's squirrel removals would not cause MGRS diet shifts; (c) evidence of dietary niche partitioning, specifically in total proportion of fungi consumed; (d) overlaps in diet proportions would be related to variability in food production; and (e) dietary niche interactions between MGRS and Abert's squirrels would be similar to those in sites of natural syntopy.

## 2 MATERIALS AND METHODS

### 2.1 Study site

We collected data from study sites in coniferous forest of 3 mountain ranges in Arizona, USA, where red squirrels and Abert's squirrels were common. We conducted experimental removals on Abert's squirrels at our primary site, Mount Graham (MG), in the Pinaleño Mountains, Graham County. This site was ≈200 ha of mixed‐conifer forest composed of Engelmann spruce (*Picea engelmannii*), Douglas‐fir (*Pseudostuga menziesii*), corkbark fir (*Abies lasiocarpa* var. *arizonica*), southwestern white pine (*Pinus strobiformis*), and ponderosa pine at 2,800–3,000‐m elevation (Hutton et al., 2003). Two sites of natural syntopy between our model species were in the San Francisco Peaks (SF), Coconino County, and the White Mountains (WM), Greenlee County. The SF site was ≈100 ha in mixed‐conifer forest dominated by Douglas‐fir and ponderosa pine at 2,500‐m elevation. The WM site was divided into 2 ≈50‐ha areas 2,800‐ and 2,400‐m elevation, dominated by Douglas‐fir and corkbark fir, and ponderosa pine and Gambel oak (*Quercus gambelii*), respectively. Red squirrel population densities at the upper and lower elevation WM sites were ≈0.3/ha and ≈0.1/ha, respectively (Derbridge & Koprowski, unpublished data). We also collected samples from introduced non‐syntopic (INS) Abert's squirrels at a ponderosa pine forest area, ≈3 km southeast from the MG site at 2,750‐m elevation, where MGRS were not present.

### 2.2 Experimental removals

**Figure 1:**
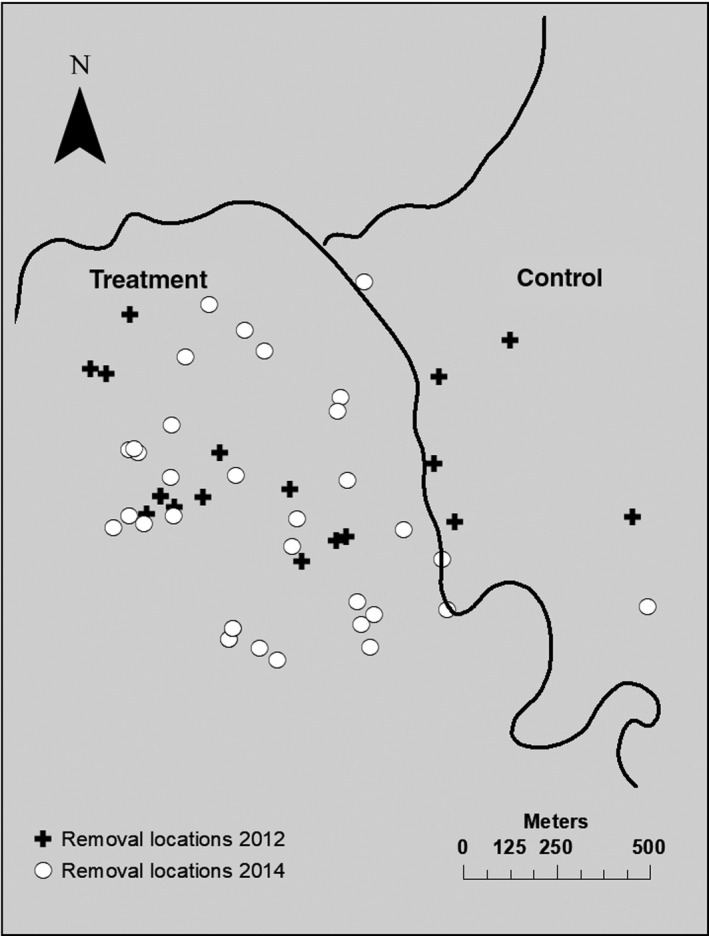
The Mount Graham study area in the Pinaleño Mountains, Arizona, USA. The black line indicates a road that bisected the study area; the Abert's squirrel treatment area was west of the road. Black crosses (2012) and white circles (2014) indicate Abert's squirrel removal locations. Animals removed from the control area were known to be primarily resident in the treatment area

**Figure 2:**
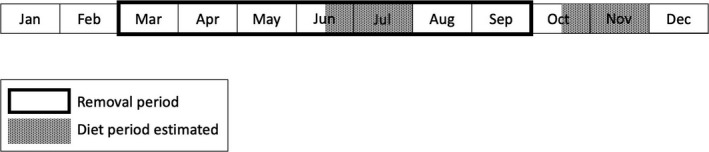
Timeline for experimental removals of Abert's squirrels in 2012 and 2014, and periods of year (based on seasonal hair growth) for which diets of Mount Graham red squirrels and Abert's squirrels were estimated. Stable isotope diet analysis used δ^13^C and δ^15^N in hairs collected from squirrels during captures and removals from the Pinaleño Mountains, Arizona, USA. Hairs collected from December to May were used to estimate fall diets. Hairs collected from August to September were used to estimate summer diets

All methods were approved by the University of Arizona Institutional Animal Care and Use Committee, US Fish and Wildlife Service, and Arizona Game and Fish Department. We used a combination of live‐trapping and hunting to remove Abert's squirrels from a 100‐ha treatment area within the MG site (Figure 1) during 2 removal periods, March‐September 2012 and 2014 (Figure 2). We captured Abert's squirrels along ≤2 traplines of ≤10 Tomahawk wire mesh live traps (custom model No. 202; Tomahawk Live Trap Company, Tomahawk, WI) spaced 25–50 m apart and euthanized them with inhalation of 100% isoflurane. We restrained captured animals in a cloth handling cone (Koprowski, 2002), and held a plastic bag containing cotton balls partially soaked with isoflurane over protruding nostrils and mouth for 30 s or until heartbeat was no longer detectable. We coordinated with local hunters to remove other Abert's squirrels and document removal effort.

We estimated efficacy of removal by surveys of Abert's squirrel feeding sign (i.e., remains of conifer cones, stripped twigs, clipped branches marked and scattered in patterns not seen in MG red squirrel sign) on 16 50‐m line transects each in the treatment and control areas. We marked transects along randomly selected azimuths taken at 16 points selected at random from a list of historically mapped points used for long‐term monitoring of the endangered MGRS. To ensure somewhat even spacing of transects, we selected 4 mapped points in each quadrant. We surveyed transects 9 times at 7‐day intervals, May–July, 2012, and 8 times at ≈14‐day intervals, May‐August 2014. We were unable to survey transects as often in 2014 due to time constraints related to concurrent studies. We also used remote cameras (Bushnell Trophy Cam model 119436; Bushnell Outdoor Products, Overland Park, KS) to establish 6 50‐m transects in each area, June‐August 2014. Two cameras faced inwards at opposite ends of the transect, and a third camera faced perpendicular to the line at 25 m. We removed vegetation that had the potential to move in wind to limit the possibility of cameras being triggered by non‐target activity. We set cameras to “Normal” sensitivity and highest image resolution (8 megapixels). As Abert's squirrels were unmarked and exclusive territories are not maintained (Farentinos, 1972), we assumed all single Abert's squirrel photographs for each hour of transect operation represented unique individuals. Where >1 Abert's squirrel occurred in a single image, we counted two animals. We used Mann‐Whitney U tests (α = 0.05) to compare counts of Abert's squirrel feeding sign and photographs between the treatment and control areas.

### 2.3 Diet sample collection and preparation

We collected known diet items of our model species from the MG site during 2011–2014, and from the SF and WM sites in 2014 and 2015, including seeds from ovulate cones of conifers (Dodd, States, & Rosenstock, 2003; Edelman & Koprowski, 2005; Froehlich, 1990), epigeous fungi (i.e., mushrooms) and hypogeous fungi (i.e., truffles and false truffles [hereafter truffles]; Keith, 1965; Smith, 1968a). We used freshly dug shallow (<50 mm) pits created by squirrels when unearthing truffles to guide our own searches, and probed the peripheries of these pits to find remaining sporocarps. We also collected conifer twigs from branches clipped by Abert's squirrels, and pollen cones from conifers at which we found squirrel feeding sign.

Both squirrel species molt in spring and fall; tails are molted only in fall (Keith, 1965; Layne, 1954). Hair contains isotopic records of diet for the period of growth. Thus, dorsal hairs collected June–September, or October–May contain isotopic diet records of about 1 month during summer and fall, respectively; tail hairs contain a fall diet record. Our hair collection regime allowed us to compare diets in non‐removal years, 2011 and 2013, with post‐removal diets in 2012 and 2014, respectively. We used Tomahawk wire mesh live traps (models #201 and custom #202; Tomahawk Live Trap Company, Tomahawk, WI) to capture squirrels and restrained them in a cloth handling cone. We collected summer‐ and fall‐growth dorsum and tail hair samples from MGRS and Abert's squirrels throughout the study, and from red squirrels and Abert's squirrels at the SF and WM sites, 2014–2015. We also collected hair samples from Abert's squirrels removed by live‐trapping or hunting during experimental removals 2012–2014, from hunted INS Abert's squirrels in 2015, and from hunted specimens at the WM and SF sites in 2014 and 2016, respectively.

We used an ultrasonic cleaner (Model FS20H, Fisher Scientific, Pittsburgh, PA) to remove coarse debris from hair samples in glass vials of deionized water. After air‐drying for 24 hr, we rinsed samples under a ventilation hood in a 2:1 chloroform/methanol solution to remove fine debris and oils (Derbridge, Krausman, & Darimont, 2012). We removed and air‐dried kernels from seed husks of conifer cones. We air‐dried fungi samples and used a scalpel to cut out clean sporocarp interiors. We used a scalpel to strip outer bark from twigs, and separately removed the exposed phloem layer. We ground diet items and hairs to powder in a Wig‐L‐Bug^®^ DS‐80 amalgamator (Crescent Dental Co., Chicago, IL). We agitated staminate cones inside a clear plastic sample bag to separate pollen from cones. We weighed 1–2 mg of all samples in a DigiWeigh DWP‐2004 laboratory scale (DigiWeigh Scales, Carlisle, PA).

We sent samples to the Environmental Isotope Laboratory (Department of Geosciences, University of Arizona) for analysis of C and N stable isotopes on a continuous‐flow gas‐ratio mass spectrometer (Finnigan Delta PlusXL) coupled to a Costech elemental analyzer. We express isotope values in delta notation (δ) as:

$$\delta Z=\left(\frac{R_{\text{sample}}}{R_{\text{standard}}}-1\right)\times 1000$$

where *Z* is ^13^C or ^15^N, and *R* is ^13^C/^12^C or ^15^N/^14^N. Standardization was based on acetanilide for elemental concentration, NBS‐22 and USGS‐24 for δ^13^C, and IAEA‐N‐1 and IAEA‐N‐2 for δ^15^N. Based on repeated internal standards, precision was better than ±0.10 for δ^13^C and ±0.2 for δ^15^N.

### 2.4 Stable isotope diet analysis

We calculated mean and standard deviation δ^13^C and δ^15^N for all diet items and squirrel species, and used *t*‐tests to determine if diet items and squirrel species were isotopically distinct (α = 0.05). Because Abert's squirrels rarely consume spruce seeds (Allred, 2010; Edelman & Koprowski, 2005), we calculated separate δ^13^C and δ^15^N that omitted spruce seeds from the Abert's squirrel diet analysis. We used a Bayesian stable isotope mixing model with rodent‐specific diet‐hair trophic discrimination factors of 3.4‰ for C and 2.4‰ for N (Kurle, Koch, Tershy, & Croll, 2014) to estimate the proportional contribution of each diet item to the fall and summer diets (Moore and Semmens 2008) of red squirrels and Abert's squirrels. We used Markov chain Monte Carlo (MCMC) methods to estimate the parameters of the mixing model, which produces simulations of plausible diet proportions and residual error consistent with the data. We ran 3 parallel MCMC chains with a burn‐in of 10,000 iterations. We generated posterior samples using 40,000 iterations of the model and a thinning rate of 10. We estimated these mixing models in R 3.4.2 (R Development Core Team 2017) and JAGS using the R package *rjags*.

### 2.5 Effect of food production on diet

**Figure 3:**
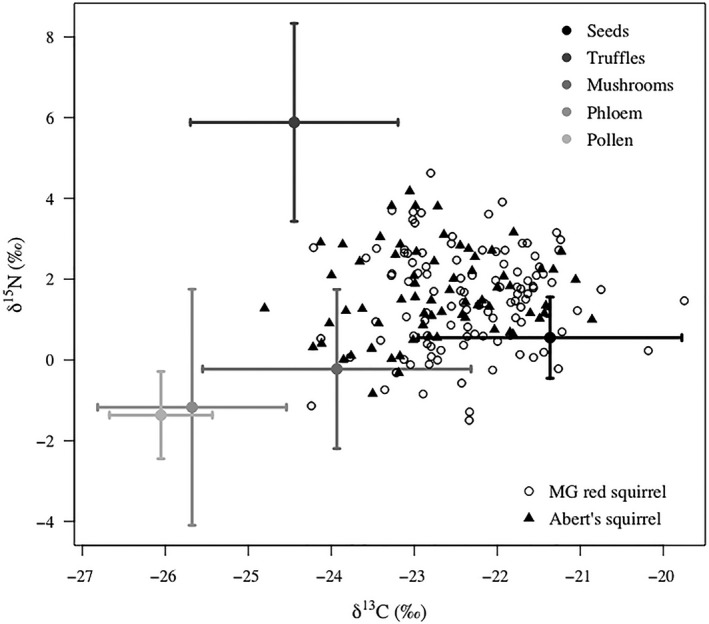
Individual δ^13^C and δ^15^N of Mount Graham red squirrels and Abert's squirrels in fall and summer, and mean and *SD* of δ^13^C and δ^15^N of their fall and summer diet items. Stable isotope values were calculated from squirrel hairs and samples of diet items collected 2011‐2014 in the Pinaleño Mountains, Arizona, USA. Mushrooms, phloem, and pollen were not isotopically distinct, and diet models used combinations of mushroom and phloem data (epphlo Figure 6) for fall diet, and mushroom and pollen data (epipol Figure 7) for summer diet

We examined the relationship between annual variation in availability of common diet items and composition of MGRS and introduced Abert's squirrel diets by taking advantage of the fact that δ^13^C of seeds and mushrooms were isotopically distinct – seeds being relatively enriched and mushrooms relatively depleted in δ^13^C (Figure 3). We used 21 years (1994–2014) of conifer seed and mushroom production data collected by the University of Arizona's MGRS Monitoring Program (Koprowski, Alanen, & Lynch, 2005) to establish long‐term food availability means and standard deviations. Conifer seeds were collected from 9 10‐m x 10‐m seed plots each containing three randomly placed 0.25‐m^2^ seed traps. Viable seeds of Engelmann spruce, Douglas‐fir, and corkbark fir were quantified as seeds/m^2^. All mushrooms of genera known to be consumed by red squirrels (Smith, 1968a, 1968b) were collected from 9 1‐m × 100‐m plots, at 2‐week intervals over 12 weeks from late July–October each year and quantified as dry weight kg/ha.

We used analysis of variance (ANOVA) to test for among‐year differences in conifer seed and mushroom production during our study, 2011–2014. We used δ^13^C as an index of the effect of variability in conifer seed and mushroom production on δ^13^C of MGRS and introduced Abert's squirrels. We used δ^13^C of individual squirrels as the response variable in linear regression models with predictors of seed and mushroom availability in each year 2011‐2014. We scaled seed and mushroom data by subtracting our 21‐year mean from each year of study and dividing by the 21‐year standard deviation. We assumed that because mushrooms and truffles formed a distinct δ^13^C group (Figure 3) and truffle production may be somewhat proportional to mushroom production (Fogel & Hunt, 1979), our model would provide insight on fungi availability in general. We included the covariates season (summer/fall), group (before/after removal), area (treatment/control), and sex. To take into account the potential effect of timing of removals on variation in δ^13^C, we included a group*area interaction in models. We used a backwards elimination process to remove non‐significant variables one at time to choose a final model by Akaike's Information Criterion (AIC) when only significant variables or the main variables of biological interest (i.e. conifer seeds and mushrooms) remained. We conducted this analysis using the *lm* function in R 3.4.2 (R Development Core Team 2017).

## 3 RESULTS

### 3.1 Experimental removals

**Figure 4:**
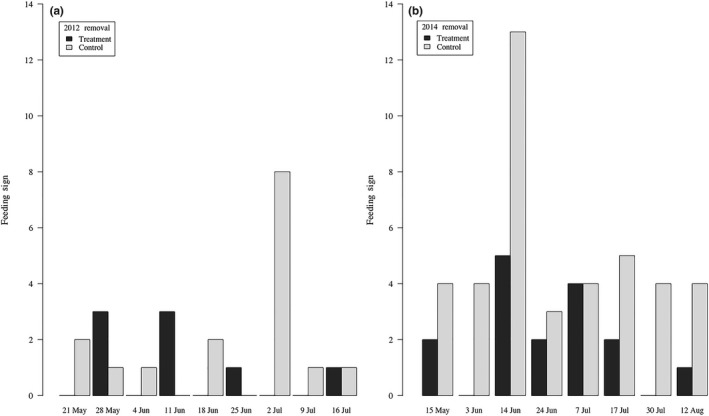
Presence of Abert's squirrel feeding sign along eight 50‐m transects in treatment and in control areas during experimental removals of Abert's squirrels between March‐September 2012 (a) and 2014 (b) in the Pinaleño Mountains, Arizona, USA

We removed 18 Abert's squirrels from the treatment area during 1,616 trap hours and 180 hunting hours 2012, and 34 Abert's squirrels from the treatment area during 2,402 trap hours and 201 hunting hours in 2014 (Figure 1). Mean presence of Abert's squirrel feeding sign was reduced in both years during removal, although not significantly between treatment ($\overset{¯}{x}=0.50$, *SD* = 1.10) and control area transects ($\overset{¯}{x}=1.00$, *SD* = 0.82) in 2012 (*W* = 29, *p* = 0.307); however, transects did differ in 2014 (*W* = 10, *p* = 0.020) with less feeding sign on treatment ($\overset{¯}{x}=1.00$, *SD* =1.32) compared to control ($\overset{¯}{x}=2.56$, *SD* = 1.55) area transects (Figure 4a, b).

**Figure 5:**
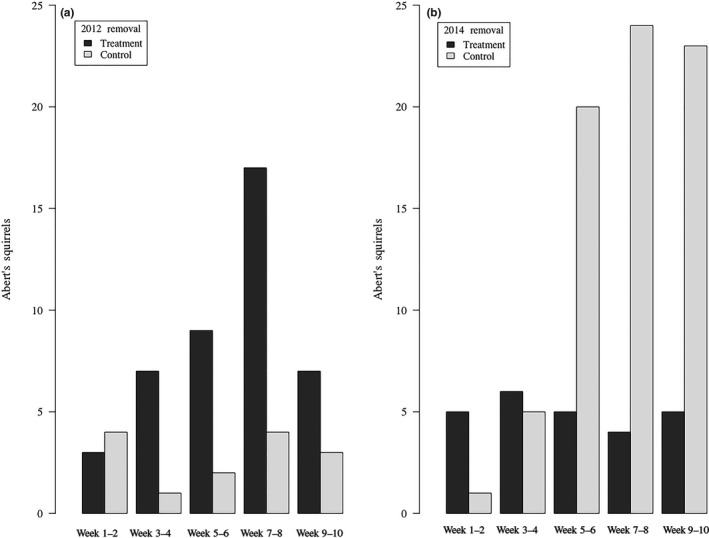
Occurrence of Abert's squirrels captured in photographs by remote cameras along 16 50‐m treatment and control area transects used to measure efficacy of experimental removals conducted March–September 2012 and 2014. Transects were operated following removals of introduced Abert's squirrels between December 2012 and February 2013 (a), and during removals June–August 2014 (a), in the Pinaleño Mountains, Arizona, USA

From ≈26,000 camera hours following the 2012 removal, we recorded 43 and 14 Abert's squirrels on the treatment and control area transects, respectively. From ≈35,000 camera hours during the 2014 removal, we recorded 25 and 73 Abert's squirrels on the treatment and control area transects, respectively (Figure 5a, b). Occurrence of Abert's squirrels on treatment ($\overset{¯}{x}=7.17$, *SD* = 4.71) compared to control ($\overset{¯}{x}=2.33$, *SD* = 1.97) transects was not different (*W* = 29, *p* = 0.091) during the winter following the first removal, and less common on treatment ($\overset{¯}{x}=4.17$, *SD* =2.32) compared to control ($\overset{¯}{x}=12.17$, *SD* = 7.47) transects during the second removal (*W* = 5.5, *p* = 0.054).

### 3.2 Stable isotope data

We collected 110 and 71 hair samples from MGRS and Abert's squirrels, respectively, at the MG site, and 7 from INS Abert's squirrels, and 47 from red and Abert's squirrels at the SF and WM sites (Table 1). From 71 samples, δ^13^C and δ^15^N of diet items were different for ≥1 isotope in most comparisons, but epigeous fungi were not isotopically distinct from phloem or pollen (Figure 3); therefore, we created two grouped diet sources from individual isotope values of epigeous fungi and phloem (i.e., epphlo), and epigeous fungi and pollen (i.e., epipol) to be used in fall and summer diet estimates, respectively. Although this grouping process limits inference on single diet items, it is recommended over discarding known diet items from analysis (Phillips et al., 2014). We commonly observed Abert's squirrels consuming pollen, and stomachs of 8 Abert's squirrels removed in May–July during the study were filled with a homogenous thick pollen‐yellow paste (Derbridge, personal observation).

**Table 1:** Mean and standard deviation of δ^13^C and δ^15^N (‰) from red squirrels, Abert's squirrels, and their diet sources at Mount Graham (MG) in the Pinaleño Mountains, San Francisco Peaks (SF) and White Mountains (WM) study areas, Arizona, USA, 2011–2016, including introduced syntopic at, and introduced non‐syntopic (INS) Abert's squirrels near the MG site

	*n*	δ^13^C		δ^15^N	
		Mean	*SD*	Mean	*SD*
MG red	110	−18.93	0.80	3.90	1.23
SF red	10	−19.41	0.57	3.51	0.76
WM red	17	−19.45	0.52	6.50	2.13
Abert's	71	−19.34	0.85	4.02	1.08
INS Abert's	7	−19.86	0.64	6.6	1.43
SF Abert's	14	−19.80	0.41	7.41	1.07
WM Abert's	6	−18.83	0.43	4.55	0.64
Red seeds	12	−21.37	1.59	0.55	1.01
Abert's seeds	6	−20.34	1.12	0.41	0.74
Truffles	10	−24.45	1.25	5.88	2.45
Mushrooms	16	−23.93	1.62	−0.23	1.97
Phloem	19	−25.68	1.14	−1.17	2.92
Pollen	8	−26.05	0.62	−1.37	1.08

Note

Separate values for seeds reflect a difference in cones targeted by red and Abert's squirrels (i.e., Abert's squirrels were not observed processing spruce cones).

### 3.3 Stable isotope diet analysis

Mount Graham red squirrel diets consisted of ≥60% conifer seeds and ≤40% fungi, pollen, and phloem in fall and summer, while Abert's squirrel diet was ≤52% conifer seeds and ≥48% fungi, pollen, and phloem at the MG site. Credible intervals for consumption of conifer seeds by introduced Abert's squirrels and MGRS did not overlap in fall or summer, and credible intervals for truffle consumption did not overlap in summer. The INS Abert's squirrels consumed a lower proportion of conifer seeds and higher proportion of truffles than MGRS and Abert's squirrels at the MG site (Table 2).

**Table 2:** Proportional contributions to diet (mean and 95% credible intervals) of 3 diet items/groups based on posterior estimates from Bayesian stable isotope mixing models of δ^13^C and δ^15^N in hairs of red and Abert's squirrels (a) in fall at Mount Graham (MG) in the Pinaleño Mountains, San Francisco Peaks (SF) and White Mountains (WM), Arizona, USA, 2011–2016, including introduced syntopic and introduced non‐syntopic (INS) Abert's squirrels near the MG site, and (b) in summer at MG, SF, and WM (Abert's squirrels were not sampled in summer at the SF, WM, or INS sites)

Squirrel_year_	*n*	(a) Fall mean (95% CI) diet estimates		
		Seeds	Truffles	Mushrooms/phloem
MGRS_2011−2014_	85	0.66 (0.60, 0.71)	0.25 (0.21, 0.29)	0.09 (0.04, 0.15)
Abert's_2011−2014_	46	0.52 (0.47, 0.57)	0.31 (0.27, 0.35)	0.17 (0.12, 0.23)
INS Abert's_2014_	7	0.27 (0.11, 0.41)	0.62 (0.46, 0.78)	0.11 (0.01, 0.28)
SF red_2014−2015_	5	0.58 (0.33, 0.83)	0.17 (0.03, 0.35)	0.25 (0.04, 0.50)
SF Abert's_2016_	14	0.27 (0.17, 0.36)	0.68 (0.59, 0.78)	0.05 (0.00, 0.14)
WM red_2014−2015_	5	0.30 (0.04, 0.59)	0.60 (0.19, 0.89)	0.10 (0.00, 0.37)
WM Abert's_2014_	6	0.55 (0.41, 0.69)	0.32 (0.20, 0.45)	0.13 (0.01, 0.29)

Squirrel_year_	*n*	(b) Summer mean (95% CI) diet estimates		
		Seeds	Truffles	Mushrooms/pollen
MGRS_2013−2014_	25	0.60 (0.49, 0.73)	0.09 (0.03, 0.16)	0.31 (0.19, 0.42)
Abert's_2011−2014_	25	0.30 (0.22, 0.38)	0.24 (0.17, 0.32)	0.46 (0.37, 0.55)
SF red_2015_	5	0.51 (0.24, 0.79)	0.19 (0.05, 0.35)	0.30 (0.05, 0.56)
WM red_2014_	12	0.42 (0.29, 0.56)	0.51 (0.36, 0.65)	0.06 (0.00, 0.20)

**Figure 6:**
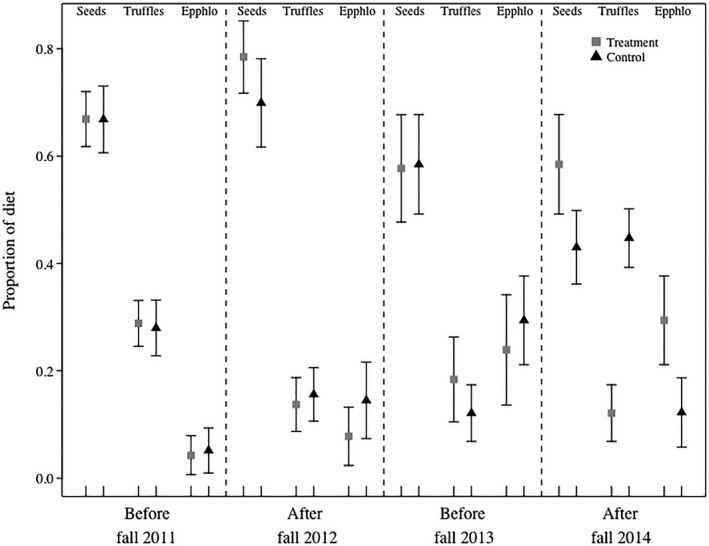
Mean and *SD* (black bars) proportional contribution of seeds, truffles, and mushrooms and phloem (i.e. epphlo) to the fall diets of Mount Graham red squirrels in treatment (squares) and control (triangles) areas before and after introduced Abert's squirrel removals in 2012 and 2014. We used a Bayesian stable isotope mixing model to estimate diet from δ^13^C and δ^15^N in hairs collected from squirrels during captures and removals in the Pinaleño Mountains, Arizona, USA

**Figure 7:**
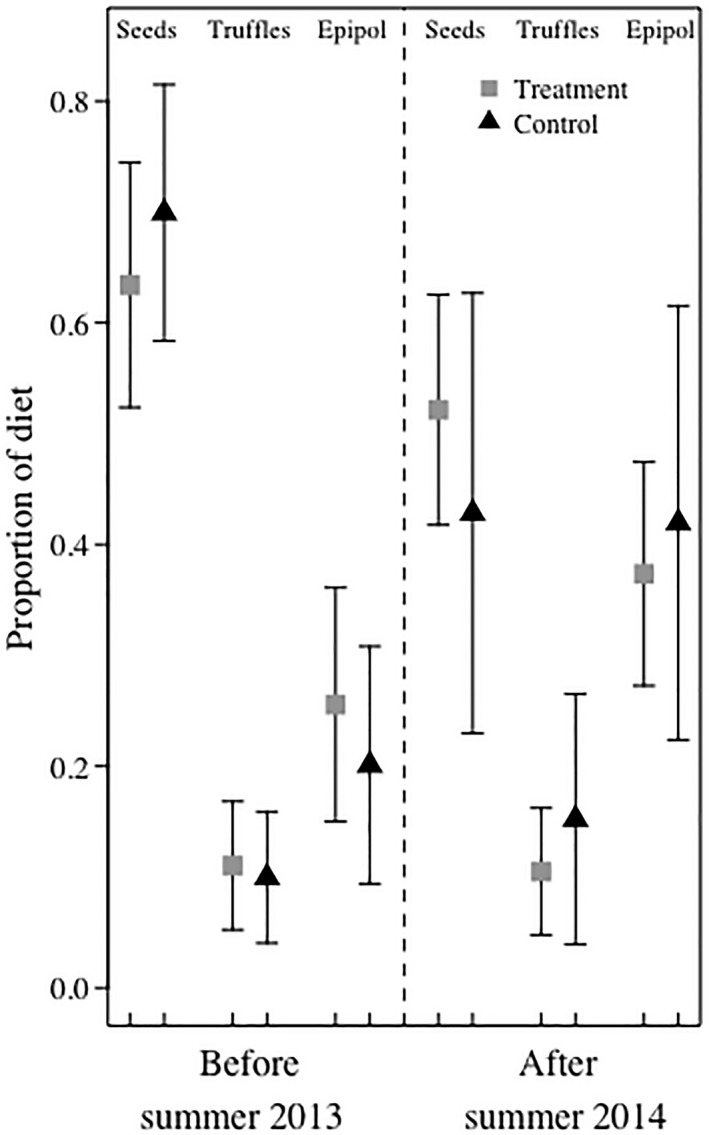
Mean and *SD* (black bars) proportional contribution of seeds, truffles, and mushrooms and pollen (i.e. epipol) to the summer diets of Mount Graham red squirrels in treatment (squares) and control (triangles) areas before and after Abert's squirrel removals in 2014. We used a Bayesian stable isotope mixing model to estimate diet from δ^13^C and δ^15^N in hairs collected from squirrels during captures and removals in the Pinaleño Mountains, Arizona, USA

The proportional contribution of all diet items to MGRS fall diet was the same in treatment and control areas before and after the first removal; all 95% credible intervals overlapped (Table 3a; Figure 6). Mean conifer seed consumption decreased in both areas after the second removal, and mean fungi consumption increased (Table 3b; Figure 6). Abert's squirrels consumed more fungi and phloem than MGRS throughout the study (Figure 6). Mount Graham red squirrel summer diet did not differ before and after the second removal; all 95% credible intervals overlapped (Table 3c; Figure 7).

**Table 3:** Proportional contributions to diet (mean and 95% credible intervals) of 3 diet items/groups based on posterior estimates from Bayesian stable isotope mixing models of δ^13^C and δ^15^N in hairs of Mount Graham red squirrels in fall before and after removal of Abert's squirrels from the Pinaleño Mountains, Arizona, USA in 2012 (a) and 2014 (b), and summer before and after removal in 2014 (c)

Period‐area	*n*	(a) Removal 1 Fall mean (95% CI) diet estimates		
		Seeds	Truffles	Mushrooms/phloem
Before‐control	9	0.67 (0.54, 0.79)	0.28 (0.18, 0.39)	0.05 (0.00, 0.16)
Before‐treatment	13	0.67 (0.57, 0.77)	0.29 (0.21, 0.37)	0.04 (0.00, 0.12)
After‐control	11	0.70 (0.54, 0.86)	0.16 (0.06, 0.26)	0.15 (0.02, 0.29)
After‐treatment	15	0.78 (0.65, 0.91)	0.14 (0.04, 0.24)	0.08 (0.00, 0.20)
		(b) Removal 2 Fall mean (95% CI) diet estimates		
Before‐control	10	0.58 (0.41, 0.77)	0.12 (0.02, 0.23)	0.30 (0.13, 0.45)
Before‐treatment	9	0.58 (0.40, 0.78)	0.18 (0.03, 0.34)	0.24 (0.05, 0.44)
After‐control	11	0.43 (0.30, 0.56)	0.45 (0.35, 0.56)	0.12 (0.01, 0.25)
After‐treatment	7	0.45 (0.26, 0.64)	0.35 (0.22, 0.50)	0.21 (0.03, 0.39)
		(c) Removal 2 Summer mean (95% CI) diet estimates		
Before‐control	5	0.70 (0.45, 0.92)	0.10 (0.01, 0.24)	0.20 (0.02, 0.43)
Before‐treatment	8	0.63 (0.42, 0.86)	0.11 (0.01, 0.23)	0.26 (0.05, 0.47)
After‐control	3	0.43 (0.06, 0.83)	0.15 (0.01, 0.43)	0.42 (0.05, 0.80)
After‐treatment	9	0.52 (0.31, 0.74)	0.10 (0.01, 0.23)	0.38 (0.17, 0.58)

Red squirrels at the SF site consumed primarily conifer seeds in fall ($\overset{¯}{x}$ = 58%) and SF Abert's squirrels consumed primarily truffles ($\overset{¯}{x}$ = 68%). At the WM site, fungi comprised ≥70% of red squirrel fall diet, and conifer seeds comprised ≥55% of WM Abert's squirrel diet. Summer diet estimates of red squirrels at the SF and WM sites followed the same pattern as fall diets (Table 2).

### 3.4 Carbon index models

From 2011‐2014, conifer seed production ($\overset{¯}{x}$ = 181.2/m^2^; *SD* = 212.1; *F*_3,32_ = 21.9; *p* < 0.001) and mushroom production ($\overset{¯}{x}$ = 5.8 kg/ha; *SD* = 5.4; *F*_3,32_ = 7.29; *p* < 0.001) differed among years. Final models for MGRS and introduced Abert's squirrels retained additional covariates, group and season, and group, sex and season, respectively (Table 4a, b). Mushroom production influenced δ^13^C of MGRS; conifer seed production influenced δ^13^C of Abert's squirrels. Season affected both species; estimates indicated MGRS δ^13^C was relatively enriched in summer, and Abert's squirrel δ^13^C was relatively depleted, suggesting availability of δ^13^C‐enriched food had a relatively greater influence on MGRS diet compared to Abert's squirrel diet (Table 5a, b).

**Table 4:** Models estimated to explain variation δ^13^C of Mount Graham red squirrels (a) and introduced Abert's squirrels (b) in the Pinaleño Mountains, Arizona, USA, 2011–2014

Model	*K*	AIC	ΔAIC	AICwt
(a) Mount Graham red squirrels				
*c + m + s + g*	6	211.35	0.00	0.54
*c + m + s + g + a*	7	212.56	1.22	0.29
*c + m + s + g + a + sx*	8	214.64	3.29	0.10
*c + m + s + g + a + sx + *(g*a)	9	215.70	4.36	0.06
(b) Abert's squirrels				
*c + m + s + g + a*	7	134.31	0.48	0.48
*c + m + s + g + a + sx*	8	135.53	0.26	0.74
*c + m + s + g*	6	136.20	0.19	0.93
*c + m + s + g + a + sx + *(g*a)	9	138.12	0.07	1.00

Notes

We used linear models with conifer seed production (*c*), mushroom production (*m*), season (*s*), group (*g*), area (*a*), sex (*sx*), and an interaction between group and area as predictors of variation in δ^13^C of Mount Graham red squirrels (*M*), and Abert's squirrels (*A*). We estimated a full model with an interaction between group and area, and removed non‐significant predictors until only the diet items and significant predictors remained. We then used AIC to select the top model.

**Table 5:** Estimates from final linear models of the effect of seed and mushroom availability on the carbon isotope values of Mount Graham red squirrels (a) and introduced Abert's squirrels (b) in the Pinaleño Mountains, Arizona, USA, 2011–2014

	Estimate	*SE*	*t‐*value	*p*‐value
(a)				
(Intercept)	−19.127	0.101	−190.113	0.000
Conifer seeds	0.230	0.143	1.608	0.111
Mushrooms	−0.845	0.134	−6.293	0.000
Group	0.449	0.171	2.622	0.010
Season	0.368	0.158	2.329	0.022
(b)				
(Intercept)	−19.605	0.155	−126.550	<0.001
Conifer seeds	0.583	0.198	2.952	0.004
Mushrooms	−0.304	0.171	−1.785	0.079
Group	0.604	0.298	2.027	0.047
Sex	0.400	0.152	2.632	0.011
Season	−0.706	0.152	−4.651	<0.001

Note

Additional covariates were included to help explain variation in the main response.

## 4 DISCUSSION

We found broad support for the hypothesis that dietary niche partitioning can facilitate coexistence between ecologically similar native and introduced species. Experimental removals of Abert's squirrels had no effect on MGRS diet, and stable isotope analysis revealed consistent differences in proportions of primary food items in diets. As predicted, consumption of fungi differed, diets were related to availability, and dietary niche interactions resembled those of the naturally co‐occurring populations at the SF and WM sites.

Our results from monitoring of removal success provided evidence that we achieved measureable population reduction. It was not possible to remove all Abert's squirrels from the treatment area and we expected recolonization to occur due to habitat connectivity and the introduced species’ ubiquity throughout forested areas of the Pinaleño Mountains. Camera data indicated Abert's squirrels were common on treatment area transects after the first removal in 2012 likely because we initiated these transects 10 weeks after concluding removals when Abert's squirrels were already recolonizing the area. During the second removal in 2014, we initiated camera transects during the peak of removal activity, and camera data revealed a clear difference in presence of Abert's squirrels in treatment and control areas. Although presence of Abert's squirrel feeding sign was statistically different only during the second removal in 2014, feeding sign was more common on control area transects during both removals. Fall diet estimates were based on δ^13^C and δ^15^N of hair grown primarily during October and early November, likely before Abert's squirrels could have recolonized sufficiently to compromise the assumption of removal.

Overall, our results demonstrate the potential for flexibility in resource partitioning according to variation in food production (Nakano et al., 1999). Introduction to the Pinaleño Mountains in the 1940s likely represented a niche opportunity (Shea & Chesson, 2002) for Abert's squirrels because high resource availability, especially at lower elevations where MGRS were rare or absent, allowed a positive rate of increase. Even in areas of syntopy with MGRS, a combination of resource availability and niche flexibility may have facilitated coexistence. Direct costs of invasion for MGRS do not appear to arise from reduced foraging opportunities, but dietary niche separation may be an ecological foundation for future competitive exclusion by the invader. To gauge the relevance for invasive species management, it will be important to understand how Abert's squirrels were able to adjust dietary niche and if further conservation implications should be expected for MGRS in competition with the introduced species.

Abert's squirrels likely achieved dietary niche separation from MGRS in several ways. First, the introduced species consumed more truffles in general but more than double the amount consumed by MGRS in summer. Second, Abert's squirrels consumed more mushrooms throughout the year (Dodd et al., 2003; States & Wettstein, 1998), but also more phloem and pollen in fall and summer, respectively. Because mushrooms were isotopically identical to phloem and pollen, we were unable to quantify their individual contributions to niche partitioning. However, phloem is a rare food item for MGRS (Froehlich, 1990) and common for Abert's squirrels (Keith, 1965), thus any proportion of phloem that provided nutrition and contributed to δ^13^C and δ^15^N of Abert's squirrels likely represented partitioning.

Abert's squirrels spend much of the earliest period of summer consuming pollen (Keith, 1965) and it may have been the most abundant food source for Abert's squirrels at the MG site before fungi production followed summer rains in early July. Climate change has caused milder winters and earlier springs in many alpine environments, and reduced snow cover along with altered phenology of diet sources favors species able to expand their latitudinal and elevational geographic ranges (Williams, Henry, & Sinclair, 2015). When introduction of exotic species to novel environments coincides with phenological changes that benefit the invader, ecologically similar native species may be displaced (Gidoin, Roques, & Boivin, 2015), and this has been demonstrated in tree squirrels (Di Febbraro, Martinoli, Russo, Preatoni, & Bertolini, 2016). Abert's squirrels have likely benefitted from increasingly milder winters during 75 years in the Pinaleño Mountains, and continuing changes to southwestern US forests (Williams et al., 2010) will likely provide further resource opportunities.

A third explanation for Abert's squirrel and MGRS dietary separation may be that they were able to partition the conifer seed component of dietary niche. Spruce cones, with thin scales and tiny seeds, and white pine cones, with coarse, resin‐drenched scales and large seeds, present foraging tradeoffs that may allow MGRS and Abert's squirrels to be superior competitors for these respective resources (Connell, 1980). Niche partitioning according to seed type or size plays an important role in community structure for many taxa including other mammals (Brown & Lieberman, 1973) and species of ants (Davidson, 1977), but if this trade‐off has been important for resource partitioning between MGRS and Abert's squirrels, the decline of spruce in the Pinaleño Mountains due to insect damage and fire (Koprowski et al., 2005) may not favor MGRS. Die‐offs of spruce (*Picea* sp.) and expansion of invasive species populations are already widespread in North American forests (Logan, Régnière, & Powell, 2003).

Although the absence of a removal effect on MGRS diet alone could not clearly demonstrate niche partitioning, our comparisons across sites were consistent with the experimental results. Red squirrels and Abert's squirrels in syntopy appear to consume different proportions of conifer seeds and fungi. This is further supported by the relative contributions of seeds to WM and SF Abert's squirrel diets. Red squirrel density was low (≈0.1/ha) where WM Abert's squirrels were sampled, but higher (≈0.3/ha; J. J. Derbridge & J. L. Koprowski, unpublished data) where SF Abert's squirrels were sampled, and the relatively low component of seeds in SF Abert's squirrel diet suggests the dietary niche of Abert's squirrels may be flexible perhaps in response to red squirrels. Exclusion of competitors by territorial species has broad implications for organization of communities. For example, in competition hierarchies of boreal ants, aggressive territorial species coexist with submissive species, but the latter species is restricted in abundance and to less‐preferred food (Savolainen & Vepsäläinen, 1988).

The hypothesis that dietary niche partitioning facilitated coexistence was further supported by our carbon index models. The greatest difference in conifer seed consumption between MGRS and Abert's squirrels occurred in 2011, when seed production was lowest during the study. Mount Graham red squirrels likely relied on seeds stored in middens during the previous year to avoid starvation following this crop failure (Smith, 1968b), but the strong influence of mushrooms represented by a negative estimate (i.e. depleted δ^13^C) in our availability model confirms this diet item's importance in a highly variable environment. When the largest cone crop during the study occurred the following year, conifer seeds contributed the largest proportions to diets of both species. The relative enrichment in δ^13^C of Abert's squirrels demonstrated the introduced species’ ability to exploit the resource opportunity of abundant seed production. The contrasting years and diet estimates suggest resource partitioning reduced competition for conifer seeds when in limited supply, and that seed production maintained the introduced Abert's squirrel population in good years. Similar patterns of coexistence based on dietary niche separation were observed among gerbils (*Gerbillus* sp.) and jerboas (*Jaculus jaculus*), and the ability of jerboas to roam widely to take advantage of patchily available resources (Brown et al., 1994) may mirror the strategy employed by Abert's squirrels that move among red squirrel territories in search of food. Dietary niche partitioning between Mount Graham red squirrels and Abert's squirrels resembled partitioning in naturally syntopic populations of these species, which suggests competition for food does not preclude coexistence. This consistent separation may be a consequence of natural selection for a system of habitat selection that tends toward minimizing competition (Rosenzweig, 1981) between red squirrels and Abert's squirrels, as has been demonstrated through removal experiments on other rodents (Abramsky & Sellah, 1982). The Abert's squirrels introduced in the 1940s were translocated from the San Francisco Peaks, one of our sites of natural syntopy, and they may have been adapted for coexistence with red squirrels. Coevolution is expected to limit niche overlap and dampen effects of competition (Smith, Mooney, & Agrawal, 2008).

Other components of ecological niche overlap may differ among populations and provide evidence MGRS are negatively affected by introduced Abert's squirrels. Niche theory predicts per‐capita growth rates to be limited by gains from resource consumption and losses from maintenance requirements, and native species could be competitively excluded if they have higher maintenance requirements than ecologically similar invaders (Shea & Chesson, 2002). In the Pinaleño Mountains, resources support invasive Abert's squirrels whose presence likely increases maintenance requirements for MGRS because they are forced to defend territories against the non‐native intruders. In addition to this potential competition deficit for MGRS, daily interactions with Abert's squirrels may cause chronic stress leading to reduced individual fitness in the endangered native species. This possibility was recently demonstrated by a removal experiment where elevated glucocorticoid concentrations in Eurasian red squirrels dropped following removal of invasive eastern gray squirrels in Lombardy, Italy (Santicchia et al., 2018).

Future studies on other components of niche overlap could help determine the potential for stable coexistence between our model species and provide insight for other invaded communities. Many of the challenges faced by MGRS are common to small populations at southern range peripheries. Such populations may be less resistant to invasion effects as they tend to possess lower genetic diversity (Vucetich & Waite, 2003) and occur in lower densities than populations at range centers (Lomolino & Channell, 1995). Mount Graham red squirrels have extremely low genetic variation (Fitak, Koprowski, & Culver, 2013) which could inhibit their potential to adapt to a changing environment. They also have much larger home ranges than other red squirrel populations (Koprowski, King, & Merrick, 2008), and low population density may increase resource availability for Abert's squirrels whose introduced population in the Pinaleño Mountains is central to the species’ native range. Rapid transformation of forests (Allen et al., 2010) and predicted declines in winter precipitation in the southwestern US (Williams et al., 2010) due to global climate change will not favor populations at southern extents of species’ ranges. Resource partitioning between ecologically similar native and introduced species may not keep pace with rapid forest ecosystem change, and the risk of competitive exclusion may be greater for vulnerable native populations.

## CONFLICT OF INTEREST

None declared.

## AUTHOR CONTRIBUTION

Both authors contributed to study design. J.D. collected and analyzed the data, and wrote the manuscript; J.K. procured funds and permits, provided edits, assisted with revisions, and gave approval for publication.

## Data Availability

The raw data relevant to all analyses in this manuscript were archived with Dryad under: https://doi.org/10.5061/dryad.966ff04.

## References

[ece35036-bib-0001] Abramsky, Z. , & Sellah, C. (1982). Competition and the role of habitat selection in *Gerbillus allenbyi *and *Meriones tristrami*: A removal experiment. Ecology, 63, 1242–1247. 10.2307/1938850

[ece35036-bib-0002] Allen, C. D. , Macalady, A. K. , Chenchouni, H. , Bachelet, D. , McDowell, N. , Vennetier, M. , … Cobb, N. (2010). A global overview of drought and heat‐induced tree mortality reveals emerging climate change risks for forests. Forest Ecology and Management, 259, 660–684. 10.1016/j.foreco.2009.09.001

[ece35036-bib-0003] Allred, S. (2010). The Natural History of Tassel‐eared Squirrels. Albuquerque, NM: University of New Mexico Press.

[ece35036-bib-0004] Bertolino, S. , Cordero di Montezemolo, N. , Preatoni, D. G. , Wauters, L. A. , & Martinoli, A. (2014). A grey future for Europe: *Sciurus carolinensis *is replacing native red squirrels in Italy. Biological Invasions, 16, 53–62. 10.1007/s10530-013-0502-3

[ece35036-bib-0005] Bovendorp, S. R. , Libardi, G. S. , Saramento, M. M. , Camargo, P. B. , & Percequillo, A. R. (2017). Age and habitat quality matters: Isotopic variation of two sympatric species of rodents in Neotropical Forest. Hystrix, 28, 214–221.

[ece35036-bib-0006] Brown, J. S. , Kotler, B. P. , & Mitchell, W. A. (1994). Foraging theory, patch use, and the structure of a Negev Desert granivore community. Ecology, 75, 2286–2300. 10.2307/1940884

[ece35036-bib-0007] Brown, J. H. , & Lieberman, G. A. (1973). Resource utilization and coexistence of seed‐eating desert rodents in sand dune habitats. Ecology, 54, 788–797. 10.2307/1935673

[ece35036-bib-0008] Chesson, P. (1994). Multispecies competition in variable environments. Theoretical Population Biology, 45, 227–276. 10.1006/tpbi.1994.1013

[ece35036-bib-0009] Chesson, P. (2000). Mechanisms of maintenance of species diversity. Annual Review of Ecology and Systematics, 31, 343–366. 10.1146/annurev.ecolsys.31.1.343

[ece35036-bib-0010] Connell, J. H. (1980). Diversity and the coevolution of competitors, or the ghost of competition past. Oikos, 35, 131–138. 10.2307/3544421

[ece35036-bib-0011] Davidson, D. W. (1977). Species diversity and community organization in desert seed‐eating ants. Ecology, 58, 711–724. 10.2307/1936208

[ece35036-bib-0012] Davis, M. A. (2003). Biotic globalization: Does competition from introduced species threaten biodiversity? AIBS Bulletin, 53, 481–489.

[ece35036-bib-0013] Davis, R. , & Brown, D. E. (1988). Documentation of the transplanting of Abert's squirrels. The Southwestern Naturalist, 33, 490–492. 10.2307/3672222

[ece35036-bib-0014] Derbridge, J. J. , Krausman, P. R. , & Darimont, C. T. (2012). Using Bayesian stable isotope mixing models to estimate wolf diet in a multi‐prey ecosystem. The Journal of Wildlife Management, 76, 1277–1289. 10.1002/jwmg.359

[ece35036-bib-0015] Di Febbraro, M. , Martinoli, A. , Russo, D. , Preatoni, D. , & Bertolini, S. (2016). Modelling the effects of climate change on the risk of invasion by alien squirrels. Hystrix, the Itaian Journal of Mammalogy, 27, 4065–8.

[ece35036-bib-0016] Dodd, N. L. , States, J. S. , & Rosenstock, S. S. (2003). Tassel‐eared squirrel population, habitat condition, and dietary relationships in north‐central Arizona. The Journal of Wildlife Management, 67, 622–633. 10.2307/3802719

[ece35036-bib-0017] Edelman, A. J. , & Koprowski, J. L. (2005). Diet and tree use of Abert's squirrels (*Sciurus aberti*) in a mixed‐conifer forest. The Southwestern Naturalist, 32, 490–492. 10.1894/0038-4909(2005)050[0461:DATUOA]2.0.CO;2

[ece35036-bib-0018] Farentinos, R. C. (1972). Social dominance and mating activity in the tassel‐eared squirrel (*Sciurus aberti ferreus*). Animal Behaviour, 20, 316–326. 10.1016/S0003-3472(72)80053-8 4674670

[ece35036-bib-0019] Ferner, J. W. (1974). Habitat relationships of *Tamiasciurus hudsonicus* and *Sciurus aberti* in the Rocky Mountains. The Southwestern Naturalist, 18, 470–473. 10.2307/3670306

[ece35036-bib-0020] Fitak, R. R. , Koprowski, J. L. , & Culver, M. (2013). Severe reduction in genetic variation in a montane isolate: The endangered Mount Graham red squirrel (*Tamiasciurus hudsonicus grahamensis*). Conservation Genetics, 14, 1233–1241. 10.1007/s10592-013-0511-x

[ece35036-bib-0021] Fogel, R. , & Hunt, G. (1979). Fungal and arboreal biomass in a western Oregon Douglas‐fir ecosystem: Distribution patterns and turnover. Canadian Journal of Forest Research, 9, 245–256. 10.1139/x79-041

[ece35036-bib-0022] Froehlich, G. F. (1990). Habitat use and life history of the Mount Graham red squirrel. MS thesis. Tucson, AZ: University of Arizona.

[ece35036-bib-0023] Gidoin, C. , Roques, L. , & Boivin, T. (2015). Linking niche theory to ecological impacts of successful invaders: Insights from resource fluctuation‐specialist herbivore interactions. Journal of Animal Ecology, 84, 396–406. 10.1111/1365-2656.12303 25318584

[ece35036-bib-0024] Gurnell, J. , Wauters, L. A. , Lurz, P. W. W. , & Tosi, G. (2004). Alien species and interspecific competition: Effects of introduced eastern grey squirrels on red squirrel population dynamics. Journal of Animal Ecology, 73, 26–37. 10.1111/j.1365-2656.2004.00791.x

[ece35036-bib-0025] Hall, J. G. (1981). A field study of the Kaibab squirrel in Grand Canyon National Park. Wildlife Monographs, 75, 3–54.

[ece35036-bib-0026] Herbold, B. , & Moyle, P. B. (1986). Introduced species and vacant niches. The American Naturalist, 128, 751–760. 10.1086/284600

[ece35036-bib-0027] Hutton, K. A. , Koprowski, J. L. , Greer, V. L. , Alanen, M. I. , Schauffert, C. A. , Young, P. J. , & Jones, C. A. (2003). Use of mixed‐conifer and spruce‐fir forests by an introduced population of Abert's squirrels (*Sciurus aberti*). The Southwestern Naturalist, 48, 257–260. 10.1894/0038-4909(2003)048<0257:UOMASF>2.0.CO;2

[ece35036-bib-0028] Keith, J. O. (1965). The Abert squirrel and its dependence on ponderosa pine. Ecology, 46, 150–163. 10.2307/1935266

[ece35036-bib-0029] Kenward, R. , & Holm, J. (1993). On the replacement of the red squirrel in Britain. A phytotoxic explanation. Proceedings of the Royal Society of London B: Biological Sciences, 251, 187–194. 10.1098/rspb.1993.0028 8097326

[ece35036-bib-0030] Koprowski, J. L. (2002). Handling tree squirrels with an efficient and safe restraint. Wildlife Society Bulletin, 30, 101–103.

[ece35036-bib-0031] Koprowski, J. L. (2005). Annual cycles in body mass and reproduction of endangered Mt. Graham red squirrels. Journal of Mammalogy, 86, 309–313. 10.1644/BWG-232.1

[ece35036-bib-0032] Koprowski, J. L. , Alanen, M. I. , & Lynch, A. M. (2005). Nowhere to run and nowhere to hide: Response of endemic Mt. Graham red squirrels to catastrophic forest damage. Biological Conservation, 126, 491–498. 10.1016/j.biocon.2005.06.028

[ece35036-bib-0033] Koprowski, J. L. , King, S. R. B. , & Merrick, M. J. (2008). Expanded home ranges in a peripheral population: space use by endangered Mt. Graham red squirrels. Endangered Species Research, 4, 227–232. 10.3354/esr00026

[ece35036-bib-0034] Kurle, C. M. , Koch, P. L. , Tershy, B. R. , & Croll, D. A. (2014). The effects of sex, tissue type, and dietary components on stable isotope discrimination factors (Δ13C and Δ15N) in mammalian omnivores. Isotopes in Environmental and Health Studies, 50, 307–321. 24787278 10.1080/10256016.2014.908872

[ece35036-bib-0035] Layne, J. N. (1954). The biology of the red squirrel, *Tamiasciurus hudsonicus loquax* (Bangs), in central New York. Ecological Monographs, 24, 227–268. 10.2307/1948465

[ece35036-bib-0036] Logan, J. A. , Régnière, J. , & Powell, J. A. (2003). Assessing the impacts of global warming on forest pest dynamics. Frontiers in Ecology and the Environment, 1, 130–137. 10.1890/1540-9295(2003)001[0130:ATIOGW]2.0.CO;2

[ece35036-bib-0037] Lomolino, M. V. , & Channell, R. (1995). Splendid isolation: Patterns of geographic range collapse in endangered mammals. Journal of Mammalogy, 76, 335–347. 10.2307/1382345

[ece35036-bib-0038] Merkle, J. A. , Polfus, J. L. , Derbridge, J. J. , & Heinemeyer, K. S. (2017). Dietary niche‐partitioning among black bears, grizzly bears and wolves in a multi‐prey ecosystem. Canadian Journal of Zoology, 95, 663–673.

[ece35036-bib-0039] Moore, J. W. , & Semmens, B. X. (2008). Incorporating uncertainty and prior information into stable isotope mixing models. Ecology Letters, 11, 470–480. 10.1111/j.1461-0248.2008.01163.x 18294213

[ece35036-bib-0040] Mooney, H. A. , & Cleland, E. E. (2001). The evolutionary impact of invasive species. Proceedings of the National Academy of Sciences, 98, 5446–5451. 10.1073/pnas.091093398 PMC33232 11344292

[ece35036-bib-0041] Nakano, S. , Fausch, K. D. , & Kitano, S. (1999). Flexible niche partitioning via a foraging mode shift: A proposed mechanism for coexistence in stream‐dwelling charrs. Journal of Animal Ecology, 68, 1079–1092. 10.1046/j.1365-2656.1999.00355.x

[ece35036-bib-0042] Nash, D. J. , & Seaman, R. N. (1977). Sciurus aberti. Mammalian Species, 80, 4065–5. 10.2307/3503958

[ece35036-bib-0043] Patton, D. R. (1974). Estimating food consumption from twigs clipped by the Abert squirrel. Rocky Mountain Forest and Range Experiment Station, USDA Forest Service.

[ece35036-bib-0044] Petren, K. , & Case, T. J. (1996). An experimental demonstration of exploitation competition in an ongoing invasion. Ecology, 77, 118–132. 10.2307/2265661

[ece35036-bib-0045] Phillips, D. L. , Inger, R. , Bearhop, S. , Jackson, A. L. , Moore, J. W. , Parnell, A. C. , … Ward, E. J. (2014). Best practices for use of stable isotope mixing models in food‐web studies. Canadian Journal of Zoology, 92, 823–835. 10.1139/cjz-2014-0127

[ece35036-bib-0046] Pulliam, H. R. (1986). Niche expansion and contraction in a variable environment. American Zoologist, 26, 71–79. 10.1093/icb/26.1.71

[ece35036-bib-0047] R Core Team . (2017). R: A language and environment for statistical computing. Vienna, Austria: R Foundation for Statistical Computing.

[ece35036-bib-0048] Rosenzweig, M. L. (1981). A theory of habitat selection. Ecology, 62, 327–335. 10.2307/1936707

[ece35036-bib-0049] Sakai, A. K. , Allendorf, F. W. , Holt, J. S. , Lodge, D. M. , Molofsky, J. , With, K. A. , … Weller, S. G. (2001). The population biology of invasive species. Annual Review of Ecology and Systematics, 32, 305–332. 10.1146/annurev.ecolsys.32.081501.114037

[ece35036-bib-0050] Santicchia, F. , van Dantzer, B. , Kesteren, F. , Palme, R. , Martinoli, A. , Ferrari, N. , & Wauters, L. A. (2018). Stress in biological invasions: Introduced invasive grey squirrels increase physiological stress in native Eurasian red squirrels. Journal of Animal Ecology, 87, 1342–1352. 10.1111/1365-2656.12853 29790583

[ece35036-bib-0051] Savolainen, R. , Vepsäläinen, K. , & Vepsalainen, K. (1988). A competition hierarchy among boreal ants: Impact on resource partitioning and community structure. Oikos, 51, 135–155. 10.2307/3565636

[ece35036-bib-0052] Sax, D. F. , & Gaines, S. D. (2008). Species invasions and extinction: The future of native biodiversity on islands. Proceedings of the National Academy of Sciences, 105, 11490–11497. 10.1073/pnas.0802290105 PMC2556416 18695231

[ece35036-bib-0053] Sax, D. , Stachowicz, J. , Brown, J. , Bruno, J. , Dawson, M. , Gaines, S. , … Mayfield, M. (2007). Ecological and evolutionary insights from species invasions. Trends in Ecology & Evolution, 22, 465–471. 10.1016/j.tree.2007.06.009 17640765

[ece35036-bib-0054] Shea, K. , & Chesson, P. (2002). Community ecology theory as a framework for biological invasions. Trends in Ecology & Evolution, 17, 170–176. 10.1016/S0169-5347(02)02495-3

[ece35036-bib-0055] Smith, C. C. (1968a). The adaptive nature of social organization in the genus of three squirrels *Tamiasciurus* . Ecological Monographs, 38, 31–64.

[ece35036-bib-0056] Smith, C. C. (1981). The indivisible niche of *Tamiasciurus *‐ an example of non‐partitioning of resources. Ecological Monographs, 51, 343–363. 10.2307/2937278

[ece35036-bib-0057] Smith, M. C. (1968b). Red squirrel responses to spruce cone failure in interior Alaska. The Journal of Wildlife Management, 32, 305–317.

[ece35036-bib-0058] Smith, R. , Mooney, K. , & Agrawal, A. (2008). Coexistence of three specialist aphids on common milkweed, *Asclepias syriaca* . Ecology, 89, 2187–2196. 18724729 10.1890/07-1441.1

[ece35036-bib-0059] States, J. , & Wettstein, P. (1998) Food habits and evolutionary relationships of the tassel‐eared squirrel (*Sciurus aberti*) In Steele M. A., Merritt J. F., & Zegers D. A. (Eds.), Ecology and Evolutionary Biology of Tree Squirrels (pp. 185–194). Martinsville, VA: Virginia Museum of Natural History Special Publication 6.

[ece35036-bib-0060] Steele, M. A. (1998). Tamiasciurus hudsonicus. Mammalian Species, 586, 4065–9. 10.2307/3504443

[ece35036-bib-0061] Vitousek, P. M. , Mooney, H. A. , Lubchenco, J. , & Melillo, J. M. (1997). Human domination of Earth's ecosystems. Science, 277, 494–499. 10.1126/science.277.5325.494

[ece35036-bib-0062] Vucetich, J. A. , & Waite, T. A. (2003). Spatial patterns of demography and genetic processes across the species' range: Null hypotheses for landscape conservation genetics. Conservation Genetics, 4, 639–645.

[ece35036-bib-0063] Wauters, L. , Gurnell, J. , Martinoli, A. , & Tosi, G. (2002). Interspecific competition between native Eurasian red squirrels and alien grey squirrels: Does resource partitioning occur? Behavioral Ecology and Sociobiology, 52, 332–341.

[ece35036-bib-0064] Williams, A. P. , Allen, C. D. , Millar, C. I. , Swetnam, T. W. , Michaelsen, J. , Still, C. J. , & Leavitt, S. W. (2010). Forest responses to increasing aridity and warmth in the southwestern United States. Proceedings of the National Academy of Sciences, 107, 21289–21294. 10.1073/pnas.0914211107 PMC3003095 21149715

[ece35036-bib-0065] Williams, C. M. , Henry, H. A. L. , & Sinclair, B. J. (2015). Cold truths: How winter drives responses of terrestrial organisms to climate change. Biological Reviews, 90, 214–235. 10.1111/brv.12105 24720862

